# Artificial neural network application for identifying risk of depression in high school students: a cross-sectional study

**DOI:** 10.1186/s12888-021-03531-5

**Published:** 2021-10-20

**Authors:** Fang-Fang Zhao

**Affiliations:** Department of Nursing Science, Faculty of Medicine, Nantong University, Nantong, 0086-226001 Jiangsu Province China

**Keywords:** Depression, Adolescents, Model, Artificial neural network

## Abstract

**Background:**

Identifying important factors contributing to depression is necessary for interrupting risk pathways to minimize adolescent depression. The study aimed to assess the prevalence of depression in high school students and develop a model for identifying risk of depression among adolescents.

**Methods:**

Cross-sectional study was conducted. A total of 1190 adolescents from two high schools in eastern China participated in the study. Artificial neurol network (ANN) was used to establish the identification model.

**Results:**

The prevalence of depression was 29.9% among the students. The model showed the top five protective and risk factors including perceived stress, life events, optimism, self-compassion and resilience. ANN model accuracy was 81.06%, with sensitivity 65.3%, specificity 88.4%, and area under the receiver operating characteristic (ROC) curves 0.846 in testing dataset.

**Conclusion:**

The ANN showed the good performance in identifying risk of depression. Promoting the protective factors and reducing the level of risk factors facilitate preventing and relieving depression.

## Background

Depression is a common syndrome in adolescents, which is decreasing quality of life and potentially leading to significant morbidity and suicidality [1]. Depression is also a leading cause of disability of adolescents worldwide [2] and accounts for global burden of disease [3]. Lifetime prevalence of depression was reported as 20% in teenagers at late adolescence [4]. The prevalence of depression is still increasing, especially among adolescents [5]. Depression in adolescence was also associated with adulthood psychosocial impairments and mental health disorders [6, 7]. Besides impairing the flourish of adolescents, suicide, the third leading cause of older adolescents’ death is the most devastating concern for adolescent depression [8]. Despite the multiple inventions and treatment, about 1/3 patients failed to achieve remission from treatment attempts [9]. Therefore, preventive measures are becoming increasing important. It is essential to timely recognize the key factors contributing to the prevalence of depression.

Many factors have been discovered to be associated with depression. Risk factors such as life events [10], and perceived stress [11] were associated with depression. Protective factors such as resilience [12], mindfulness [13], self-efficacy [14] and social support [15, 16] were inversely associated with depression.

Although studies have tended to focus on correlation of some risk factors and protective factors with depression separately, the key important factors in analyzing these factors at the same time remain poorly understood. Little is known about which factors in combination of environmental factors and individual factors mostly influencing depression. A targeted prevention strategy is suggested for its effective efforts in preventing the development of depression in adolescents at risk [7]. Prevention measures could target at reducing the modifiable risk factors and improving levels of protective factors for adolescents at high-risk. Preventions targeting at high-risk groups are promising. But the key components for these strategies and programs remain unclear and need to pay priority attention in future depression research [7]. Therefore, identifying the key factors from a variety of factors and the students at risk of depression are essential for prevention and treatment of depression.

In addition, self-compassion was inversely associated with psychological distress [17]. Other factors like exercise, nutrition diet, anxious responses when facing examinations, having a clear goal, and optimism were unsure whether to be the significant factors contributing to depression among adolescents. Exercise is beneficial for mental health and may protect against depression [18]. However, no clear evidence indicated that all levels of exercise contributing to increasing positive mental health and decreasing negative health. The present study will examine whether exercise is an important factor related to depression. Examinations are of major importance for high school students and may cause anxious responses if they cannot handle. In China, parents have high expectations for children’s academic performance. Thus, this social pressure may stimulate their anxiety for exams. Optimism involves positive expectations and attitude that outcomes will be in the right direction and desirable [19]. Optimism has been associated with coping skills [19]. Depression onset was predicted by hopelessness in adolescents [20]. Therefore, low level of optimism is potentially related to depression among adolescents. Having a clear goal for future means students may focus on their study goal with energy and have less time indulge in emotional worries. This distraction may be a protective factor for depression [21]. Considerable interest has been gained in the association between nutrition and mental health. Poor nutrition may be associated with low mood [22].

How these protective factors and risk factors mentioned above functions together on depression onset is not clear. Identifying important factors contributing to depression is necessary for interrupting risk pathways to minimize adolescent depression. Identifying the important factors from potential related factors may offer more precise components to target and modify in interventions.

An increasing interest has been in the trend in developing models for identifying the risks of depression. In this study, artificial neural network was used to identify depression and which factors are among the highest contributing features. Artificial neural networks (ANN) are computer models that imitated human brain, which can process multidimensionality of complex data and detect all possible interactions in building predictive or identification model.

The study aimed to identify the key factors from demographics, individual factors and environmental factors that may contribute to the depression of students and detect the pattern of depressive students.

## Methods

### Study design

A cross-sectional design was employed and machine learning method was used to examine the factors associated with depression among high school students.

**Fig. 1:**
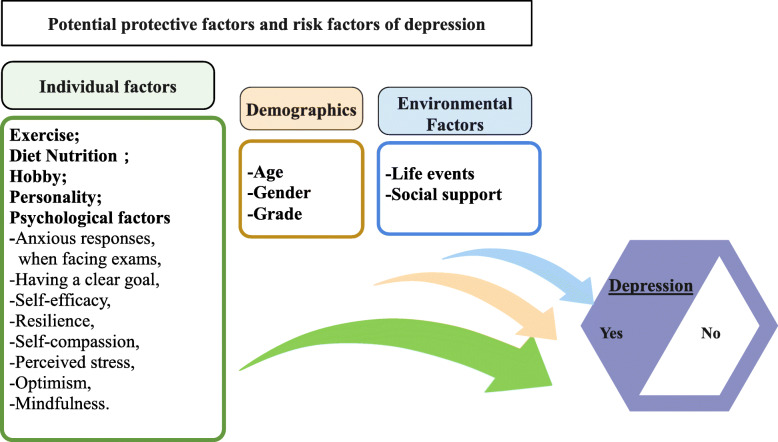
Hypothesized potential protective factors and risk factors of depression in the study

Protective and risk factors (Input variables) include 1) Demographics (age, gender and study year); 2) Individual factors (Exercise; hobby; diet nutrition, personality; psychological factors including anxious responses when facing exams, having a clear goal, self-efficacy, resilience, self-compassion, perceived stress, optimism, and mindfulness). 3) Environmental factors (Life events, social support). Figure 1.

### Participants

Participants were high school students from two middle schools in Qingdao, Shandong Province. Inclusion criteria were that participants were high school students and willing to participate after instruction. The entrance age for the first year of high school in China was usually 15 years old. Exclusion criteria of recruitment were that students who were not high school students or younger than 14 years. The researcher first explained the study aim and methods to the teachers of the high schools. Students were recruited using convenient sampling via teachers or head of teachers in the two high schools. The students were given the internet link and scanning code generated by online tool (questionnaire star, Changsha Ranxing information technology Co, Ltd., China) to read the instruction of the study aim and procedure in detail again before deciding participation or not.

### Ethical considerations

The study conformed to declaration of Helsinki and obtained the approval from the Ethical review of a University in China (2019–17/2020–21). The potential participants read the aims and instructions of filling questionnaires, and informed their parents. If they agreed to participate after reading the instruction, they could discuss with their parents at home during holiday and made the final decision to completed the questionnaires or not. They can click quitting the questionnaire link at any time and if they agree they continue to fill the questionnaires. The participation was totally voluntary. They can withdraw at any time. The instruments in the study were approved by original authors when necessary.

### Measures for input and output variables

#### Demographics and single-item measures

Demographics included age, gender and grade (study year). Exercise such as jogging and playing balls daily was measured by a question responded as follows: over an hour (scored 1), 0.5-1 h (scored 2) and below 0.5 h (scored 3). Hobby was measured by a question “Whether you had a wide range of hobbies”, with responded as follows: Not wide at all (scored 1), not wide (scored 2), not sure (scored 3), wide (scored 4), very wide (scored 5). Diet nutrition was measured by a question with responded choices as follows: food types were various, nutrition was balanced (Scored 1); food types and nutrition were in general level (Scored 2); food types are limited and nutrition was not balanced (Scored 3). Anxious responses when facing exams were measured by a question responded as follows: very anxious (scored 1), anxious (scored 2), not sure (scored 3), a bit anxious (scored 4), no anxious at all (scored 5). Having a clear goal for future was measured by an item responded as follows: None (scored 1), largely not (scored 2), sometimes (scored 3), yes (scored 4).

#### Multidimensional scale of perceived social support (MSPSS)

Social support was measured by MSPSS [23]). The scale has three dimensions named family, friends and significant other. The MSPSS has 12 items with 7 possible responses to each statement, scored from 0 to 6. The higher score indicates a higher level of MSPSS. Cronbach’s a was 0.88 [24].

#### Adolescent self-rating negative life events checklist

Life events were measured by Adolescent self-rating life events checklist, including if the negative life events happen and their negative influence over the past 12 months [25]. The scale has 26 coding items with two kinds of response options [26]. Each item represents a negative life event such as misunderstanding by others, failed in examination, conflict with classmates, tense relationships with teachers, accident, disease, family economic difficulties. If the life events did not happen, the answer was scored 0. If happened, the negative influence (scored from 1 indicating no influence at all to 5 indicating extremely serious) will be chosen. The Cronbach’s alpha was 0.92, and it had good validity [26]. Higher scores indicate more serious negative influence.

#### Perceived stress scale (PSS)

Perceived stress was measured by Perceived Stress Scale [27]. The PSS consists of 10 items measuring the experience of stressful situations in the past month. The scale has response options from never (0) to very often (4). Four items are reversing responses. A higher score means higher level of perceived stress. Cronbach’s α of the scale is good and was 0.86 [28].

#### 10-item Connor-Davidson resilience scale

Resilience was measured using the 10-item Connor-Davidson Resilience Scale [29], which is modified from original scale [30]. The scale has a dimension with 5-likert responses from never (0) to almost always (4). The Cronbach’s a was 0.85 [29]. The higher score means a higher level of resilience.

#### Generalized self-efficacy scale

Self-efficacy was measured by Generalized Self-Efficacy Scale, which consists 10 items [31]. Cronbach’s alphas ranged from 0.79 to 0.90 [32]. The four-point responses are from not at all true (1) to exactly true (4). A higher score indicates higher self-efficacy.

#### Neff’s self-compassion scale (short-form)

Self-compassion was measured by Neff’s Self-Compassion Scale (Short-form) [33]. The short version scale consists of 12 items and 6 dimensions as follows: self-kindness, common humanity, mindfulness, self-judgment, isolation, over-identified. The response options were from almost never (1) to almost always (5) [34]. Cronbach’s alpha was over 0.82 [34, 35]. The total score was summing the positive items and negative items after reversing. The higher score indicates high level of self-compassion. Although self-compassion scale has the mindfulness component, it is “…narrower in scope than mindfulness more generally” [36] (p20). The general and broader concept of mindfulness includes all awareness of all aspects of experience. Therefore, using independent measures to test self-compassion and mindfulness was suggested [17].

#### Five facet mindfulness questionnaire (FFMQ)

Mindfulness was measured by Five Facet Mindfulness Questionnaire (FFMQ) [37]. The scale consists of 39 items and five dimensions including observing, describing, acting with awareness, nonjudging of inner experience, and nonreactivity to inner experience. The response options were ranged from never or very rarely true (1) to very often or always true (5). Cronbach’s a of five facets ranged from 0.75 to 0.91 [37]. FFMQ total scores are obtained by summing reversing responses and positively stated items. A higher score indicates higher level of mindfulness.

#### 10- item personality measure

Personality was measured by 10-item Personality Measure. The scale is a measure of the Big Five (or Five-Factor Model) dimensions including extraversion, agreeableness, conscientiousness, emotional stability and openness to experiences [38]. Response options ranged from disagree strongly (1) to agree strongly (7). A higher score indicates higher level of extraversion, agreeableness, conscientiousness, emotional stability or openness to experience. Total scores are obtained by summing reversing responses and positively stated items. Test–retest reliability averaged 0.72 for the 5 dimensions [38].

#### Life orientation test (LOT)

Optimism was measured by Life Orientation Test (LOT) [19]. The LOT-R is shortened from LOT [39] which has 10 items. Fillers includes items 2, 5, 6, and 8. The response options ranged from strongly disagree (0) to strongly agree (4). The Cronbach’s a was 0.74 [40]. Total score was summing by positive and negative items after reversing. High values imply high level of optimism.

#### Patient health questionnaire (PHQ)

Target variable depression was measured by PHQ-9, the patient health questionnaire. The PHQ-9 consists of nine symptom items of depressive in the past 2 weeks. Response options ranged from “not at all (scored 0),” to “nearly every day (scored 3)”. The sum scores ranged from 0 to 27. The scores indicating severity of depressive symptoms was categorized as follows: 0, none; 1–4, minimal; 5–9, mild; 5–9; moderate, 10–14; moderately severe, 15–19; or 20–27, severe. PHQ-9 ≥ 10 was found to have a sensitivity of 88% and a specificity of 88% to detect major depression, with MHP (mental health professional) reinterview as criteria [41]. Thus, PHQ-9 scores of ≥10was classified as major depression in this study. The PHQ-9 has Cronbach’s α > 0.8 and has been validated in different groups [41, 42].

### Data collection

Data were collected by online tool, questionnaire star during the holiday of National Day from 2020 October 1st to 2020 October 9th. The survey was gained the teachers’ informed consent and questionnaires with instruction of the study aim and procedure were distributed by teachers and the participation was informed totally voluntary at school. The online questionnaires were distributed on national holiday when students were at home for holiday. Thus, students had sufficient time to discuss with their parents. With parental consent, they freely chose to fill the questionnaires or not.

### Data analysis

IBM SPSS Modeler Version 18.2.2 and IBM SPSS 25.0 were used to analyze the data. The data were cleaned by imputing the missing values in age, replacing the outliers with the ones closed to normal ones and discarding the extremes. Outliers exceed the range of Q1–1.5 × IQR (Interquartile range) and Q3 + 1.5 × IQR. Extremes exceed the range of Q1–3 × IQR and Q3 + 3 × IQR. Outliers were replaced by the normal values closest to them: Q1–1.5 × IQR (Interquartile range) or Q3 + 1.5 × IQR. The action of coercing outliers/discarding extremes was conducted in the SPSS modeler. Median was used as imputation method of missing value, which replaces missing data with median of non-missing elements in the corresponding variable. Fifty-one missing values in age were imputed by the median of the ages in same grade in the study. The cleaned data were standardized by subtracting the mean score and divided by standard deviation, so that all the continuous inputs are at a comparable range. The averaged overall accuracy and AUC (area under the ROC curve) of ANN model, decision tree, Bayesian networks and logistic regression were chosen to select the better one for identifying the risk of depression in SPSS modeler setting with five-fold cross validation. The dataset was then divided into two datasets in division node: Training set (70%) and Testing set (30%). The model built and patterns extraction is conducted in training dataset and evaluated in the testing dataset.

ANN has the capability to process the nonlinear relationships between input and output via a training dataset, when traditional approaches are insufficient to calculate the complex input-output relationship [43]. After the training process by ANN, the ANN was validated with the testing dataset and the performance ranked with the other machine learning methods including decision tree, logistic regression, and Bayesian network according to overall accuracy and area under the ROC curve (AUC). Decision trees is a classification technique that can identify efficiently the most important contributing factors. It develops a predictive algorithm to identify the factors differentiating the sample population on a target variable, until it reaches one of the terminal nodes, the percentage of each class in the terminal node was provided [44]. The decision tree algorithms include C5.0, C&R Tree (Classification and Regression tree), CHAID (Chi-Square Automatic Interaction Detection) and Quest. Bayesian networks can model variables simultaneously in an exploratory manner. Logistic regression models the linear relationships of a set of independent variables and dependent variables and the occurring probability of the dependent variable is determined by the estimated probability.

Model performance were evaluated by accuracy (The percentage of correct identifications in total identifications.), sensitivity, specificity and receiver operating characteristic (ROC), area under the ROC curve (AUC). The sensitivity is true positive rate and reflects the percentage of depressive students that are correctly identified in the model. Specificity is the true negative rate and reflects the percentage of students who are not depressed that are correctly identified in the model. ROC analysis estimates a curve that describes the inherent trade-off between sensitivity and specificity for prediction or identification tool. AUC is an important metric to evaluate prediction or identification tools ranged from 0 to 1, with 1.0 reflecting perfect discrimination and 0.5 reflecting that the discrimination is by chance alone [45]. To test the generalization ability of the ANN model, ten-fold cross validation was used.

**Fig. 2:**
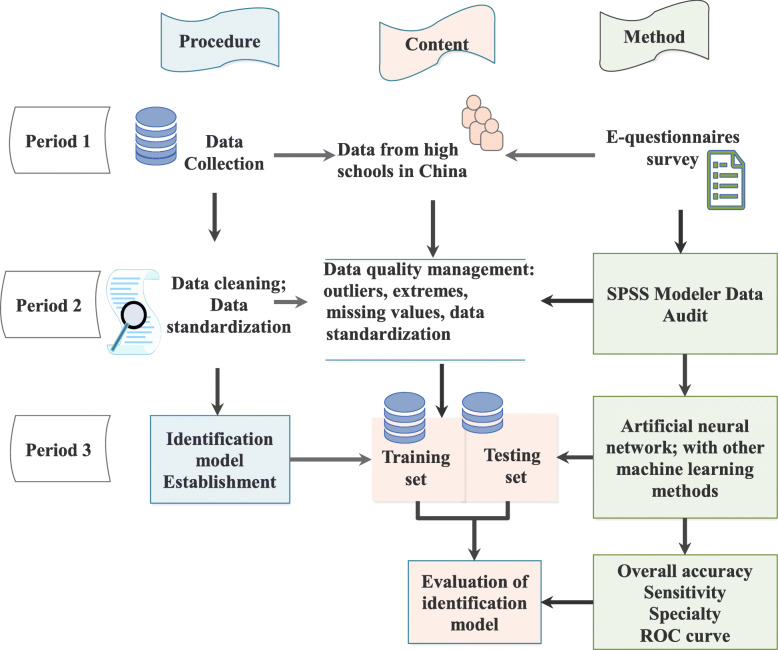
The flowchart of the study

The procedure and method of data collection and analysis were shown in Fig. 2.

## Results

A total of 1190 students participated in the e-survey. The completion time of the questionnaires in the back-stage management system of the online survey tool (questionnaire star) were checked by the researcher. Among them, 115 quick click ones were excluded based on the much shorter completion time compared to others during the survey. Thus, 1075 questionnaires were retained for analysis. The fifty-one missing values in the age variable were imputed by age median of the same grade. There were outliers and/or extremes in variables including perceived stress, social support, personality, life events, resilience, self-compassion, optimism and mindfulness. Among them, entire observations with extremes were deleted. After coercing outliers and discarding extremes conducted in the SPSS modeler, 1029 students’ questionnaires were included in the study.

### Demographics and compassion between groups

The girls accounted for 59.2% and boys amounted for 40.8%. The prevalence of depression was 29.9% among the students in the study. For girls, the prevalence of depression was 28.4% and for boys was 32.1% in the study. The detailed descriptive information for study variables, statistical test results for the factors between two groups with depression or not were shown in Table 1.

**Table 1:** Input variables and the difference between no depression group and depression group

Input variables	Total number		Depression groups		x^2^/t	p
	Mean Std.de/n (%)	Range in scale	No (*N* = 721;70.1%)	Yes (*N* = 308;29.9%)		
			(Mean Std.de/ n (%)	(Mean Std.de/ n (%)		
**Demographics**						
Age	16.06 (0.74)	14–18	16.07 (0.75)	16.03 (0.73)	0.675	*p* > 0.05
Gender					1.654	*p* > 0.05
Male	420 (40.8%)	NA	285 (39.5%)	135 (43.8%)		
Female	609 (59.2%)	NA	436 (60.5%)	173 (56.2%)		
Grade					0.774	*p* > 0.05
First year	495 (48.1%)	NA	352 (48.8%)	143 (46.4%)		
Second year	480 (46.6%)	NA	330 (45.8%)	150 (48.7%)		
Third year	54 (5.3%)	NA	39 (5.4%)	15 (4.9%)		
**Individual factors**						
Hobby	3.60 (1.04)	1–5	3.64 (1.01)	3.50 (1.09)	2.047	*p*<0.05
Exercise	1.62 (0.63)	1–3	1.59 (0.61)	1.69 (0.66)	−2.274	*p*<0.05
Diet nutrition	1.36 (0.53)	1–3	1.29 (0.49)	1.52 (0.59)	−5.992	*p*<0.001
Personality	44.46 (7.29)	27–63	46.04 (7.45)	40.78 (5.34)	12.774	*p*<0.001
**Psychological factors**						
-I Have a clear goal for future	3.18 (0.61)	1–4	3.24 (0.57)	3.03 (0.66)	4.987	*p*<0.001
-Anxious responses when facing exams	3.51 (1.09)	1–5	3.61 (1.03)	3.26 (1.18)	4.566	*p*<0.001
-Self-efficacy	28.46 (6.92)	10–40	29.00 (6.79)	27.19 (7.06)	3.883	*p*<0.001
-Self-compassion	38.04 (3.85)	30–46	39.00 (3.84)	35.80 (2.81)	13.150	*p*<0.001
-Mindfulness	119.47 (6.61)	106–134	120.50 (6.84)	117.07 (5.33)	8.655	*p*<0.001
-Resilience	28.14 (7.84)	4–40	29.35 (7.41)	25.31 (8.08)	7.523	*p*<0.001
-Perceived stress	17.71 (4.38)	8–28	16.35 (4.14)	20.88 (3.07)	−19.404	*p*<0.001
-Optimism	13.09 (2.36)	8–20	13.59 (2.38)	11.95 (1.89)	11.774	*p*<0.001
**Environmental factors**						*p*<0.001
Social support	51.90 (14.70)	9–72	54.13 (13.57)	46.67 (15.88)	7.193	*p*<0.001
Life events	31.56 (27.96)	0–111	24.58 (24.01)	47.91 (29.73)	−12.180	*p*<0.001

Note. Std.de, standardized deviation. N, the total number of participants in the groups; n, number in the subgroups. NA, not applicable

### Model selection and artificial neural network (ANN) analysis

The auto classifier estimated and ranked algorithms. The averaged overall accuracy, with AUC which was an index of performance of models. Accuracy and AUC of ANN (78.81%, 0.834), logistic regression (77.84%, 0.843), CHAID (76.95%, 0.803), C5.0 (76.38%, 0.755) Quest (75.82%, 0.663), C&T (75.66%, 0.767), and Bayesian networks (72.65%, 0.773) were ranked with five-fold cross-validation. The performance of ANN is better to identify depression. ANN model was selected for identifying risk of depression in the study.

Then, the 70% of the dataset was used as training set and 30% of the dataset was used as testing set. Artificial Neural network was conducted with inputs, hidden neurons and outputs. The structure of the selected ANN has one input layer, one hidden layer and one output layer. These inputs include demographics (age, gender, grade), and individual factors (hobby, exercise, diet nutrition, personality), psychological factors (anxious responses in facing exam, have a clear goal for future, self-efficacy, resilience, self-compassion, perceived stress, optimism, and mindfulness), and environmental factors (life events, social support). Each neuron processes the inputs using a non-linear transfer function (Customer size of unit hidden layers was set 8). A multi-layer perception (MLP) was used to train the model. The identification of the risks of depression was thought to be a form of pattern recognition and the ANN was trained using patterned function which classifies inputs into a target.

### Evaluation of the ANN model

In the training set (*n* = 728), overall accuracy of the ANN was 81.59%, sensitivity was 57.3%, specialty was 91.7%, AUC was 0.871.

After the training process, the network was tested with the remaining students (*n* = 301) that were not randomly chosen for training. Based on the results in the testing set, ANN had a higher proportion of correctly identified students who have depression (Sensitivity) than CHAID, Quest, C5.0, C&T Tree, Logistic regression and Bayesian Network, and ANN model had lower proportion of correctly identified students who have no depression (Specificity) compared to Quest in this study. However, ANN model had better accuracy and discriminating power with an AUC of 0.846 among all the tested models. Table 2.

**Table 2:** Comparison of predictive performance of ANN, CHAID, Quest, C5.0, C&T Tree, Logistic regression, and Bayesian Network of testing set (*N* = 301)

	ANN	Logistic regression	Quest	C&T Tree	C5.0	CHAID	Bayesian Network
Accuracy (%)	81.06	80.07	78.41	77.74	76.41	73.42	70.43
Sensitivity (%)	65.3	64.2	46.3	62.1	54.7	64.2	55.7
Specificity (%)	88.4	87.4	93.2	85.0	86.4	77.7	80.7
Area under ROC curve (AUC)	0.846	0.843	0.719	0.783	0.773	0.806	0.711

Note: ANN, artificial neural network. CHAID, Quest, C5.0 and C&T Tree are decision tree algorithms. C&R Tree, Classification and Regression tree, CHAID, Chi-Square Automatic Interaction Detection

### Factor importance in ANN model

**Fig. 3:**
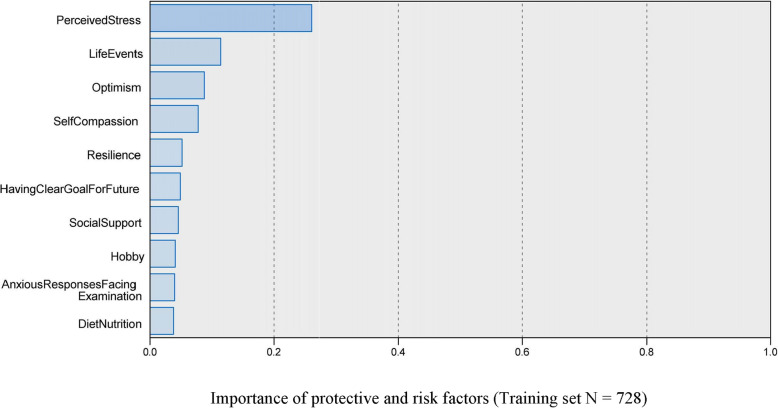
Importance of protective and risk factors (Training set *N* = 728)

**Fig. 4:**
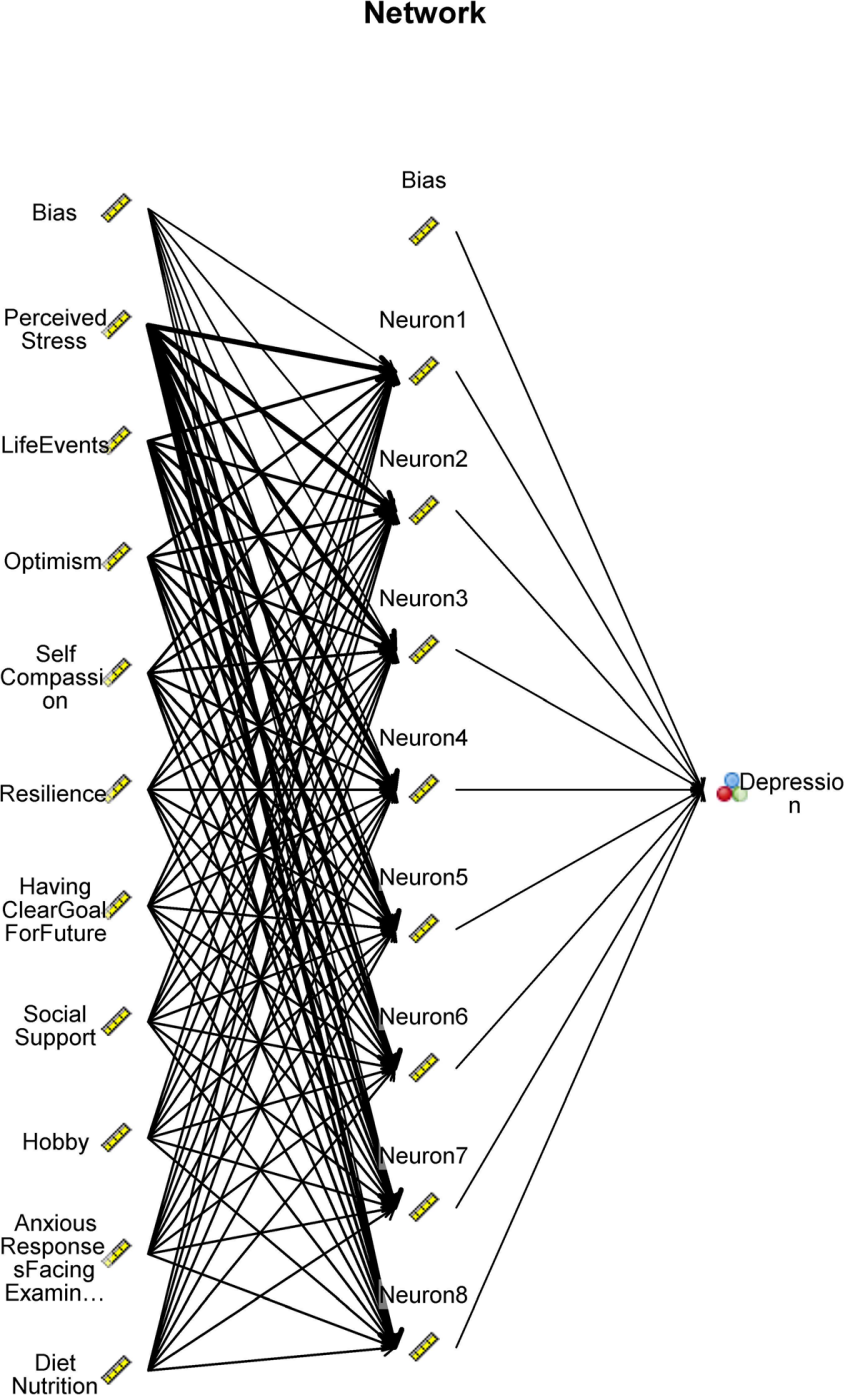
The effects of the factors in artificial neural network

Figure 3 showed that the ANN model recognized 10 important factors for the network to identify the depression of the students. The 10 important with the normalized coefficients were as follows in order: perceived stress (importance = 0.261), life events (importance = 0.114), optimism (importance = 0.087), self-compassion (importance = 0.077), resilience (importance = 0.051), having clear goal for future (importance = 0.049), social support (importance = 0.045), hobby (importance = 0.040), anxious responses when facing exams (importance =  0.039), diet nutrition (importance = 0.038). Figure 3 shows the importance of each factor in the algorithms. Figure 4 showed the ANN model with the effects of top 10 important factors on identifying depression through the eight neurons’ processing by a non-linear transfer function in hidden layer.

### Overall accuracy and coincidence matrix of ANN model

Overall accuracy of the ANN was 81.59% in training set and 81.06% in testing set. AUC was 0.871 in training set and 0.846 in testing set.

The coincidence matrix in training set and testing set was shown in Table 3. It indicates actual and the identified value of the model for depression. True positive (TP) indicates the model identifying the actual depression. True negative (TN) indicates the model identifying actual state of not depression. False positive (FP) and false negative (FN) indicates the model identifying incorrectly.

**Table 3:** Coincidence matrix of ANN

Algorithm		Predictive value							
		Training set (*N* = 728)				Testing set (*N* = 301)			
			Not Depression	Depression	Total		Not Depression	Depression	Total
ANN	Actual Value	Not Depression	472 ^TN^	43 ^FP^	515	Not Depression	182 ^TN^	24 ^FP^	206
		Depression	91 ^FN^	122 ^TP^	213	Depression	33 ^FN^	62 ^TP^	95

Note:TN, true negative; TP. True positive; FN, false negative; FP, false positive

### Sensitivity, specificity and receiver operating characteristics (ROC) in training set and testing set of ANN model

The sensitivity was 57.3% in training set and 65.3% in testing set. The specificity was 91.7% in training set and 88.4% in testing set. Although the sensitivity was not high, considering the percentage of depression was 29.9%, the sensitivity was acceptable.

**Fig. 5:**
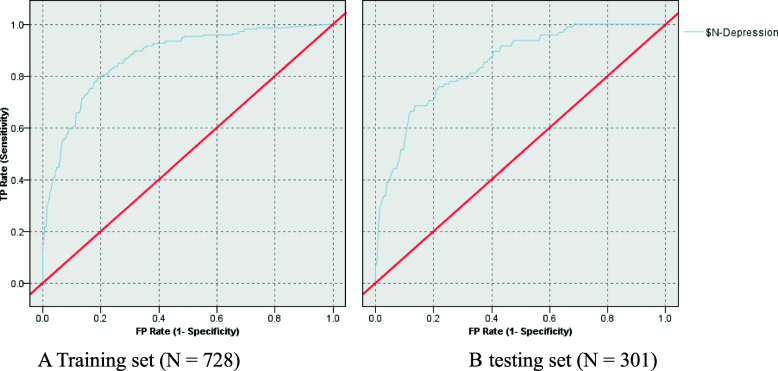
**A, B**. Chart of area under the receiver operating characteristic (ROC) curves ROC curves for the ANN model. Training set (*N* = 728). b testing set (*N* = 301)

ROC chart is shown in Fig. 5A,B. The vertical axis represents sensitivity and horizontal axes represents 1-specificity. The area under the curve (AUC) was 0.871 in training dataset and 0.846 in testing dataset.

### Ten-fold cross validation

The averaged overall accuracy was 77.90% and AUC was 0.830 in the ten-fold cross validation of ANN model. The results were close to those that had the partition of 70% training set and 30% testing set in accuracy and AUC, which showed that ANN model identification was stable and had generalization ability.

## Discussion

In the 17 factors included in analysis, the top 10 ones were perceived stress, life events, optimism, self-compassion and resilience, having clear goal for future, social support, hobby, anxious response when facing exams, and diet nutrition. The ANN model showed better performance in depression identification, which had 0.871 AUC in the training dataset and 0.846 AUC in testing dataset.

Perceived stress is the most highly influencing factor of depression among high school students in the present study. Perceived stress is a distinct construct from depression and anxiety [27], and different from life events. Life events are the stressors, and the present study also confirmed the relationships between negative life events and depression in consistent with previous study [10]. Adolescents are more vulnerable to the influence of stressors and stress perception because of their developing brains [46]. But there are other positive psychological resources exerting important influence on mental health in this present study and can buffer the negative effect of stressors.

Optimism is an important protective factor of depression among adolescents in the present study. Students with low level of optimism were more likely to have depression. Optimism has been indicated to be associated with individuals’ physical and mental health [47]. In the present study, optimism is essential in decreasing or preventing depression among adolescents. This result may be explained that optimistic individuals are more likely to adapt coping strategies in face of problems [48], more likely to adjusting negative emotion. Developing the students’ positive attitude and perspectives toward negative situation is beneficial in preventing depression.

Self-compassion, includes self-kindness, common humanity, and mindfulness, is the ability to be gentleness with oneself when facing challenge or failure. Self-kindness during difficult times is different from self-indulgence or decreasing standards [49]. Self-compassion was important in decreasing the risk of depression in high school students in this study. Individuals with higher level of self-compassion tend to display self-kindness to relive their suffering and make healthy changes to improve themselves [50]. In addition, self-compassion enables individuals to accept themselves unconditionally and treat themselves kindly which can reduce the negative impact by stress [35].

Resilience refers to the individuals’ ability to navigate towards health-maintaining resources in the face of adversity, and the capacity to bounce back from adverse events [51]. The present study indicated its importance role in defensing the risk of depression, and support the finding that resilient persons could better cope with adversity and exhibited fewer mental problems [52]. Although mindfulness variable did not appear in top 10 importance factors in identifying depression, mindfulness techniques were suggested to be an effective way to train resilience [53], which in turn, negatively associated with depression.

Having a clear goal may be beneficial for focusing attention on the pursuit of life goal and less time to indulging in negative emotion. It is suggested that interventions that specifically address goal pursuit are more likely to decrease depressive symptoms in clinical and non-clinical populations [54]. Having a clear goal is the first step before goal pursuit. Teachers and parents may be good supporters to help adolescents to set a clear future goal to pursuit.

The present study also showed social support was important in defensing depression. The support sources varied across life periods. Family members are the most important support among children and adolescents, while spouses are the main support among adults and elder adults, followed by family and then friends [15]. Social support is an environmental protective factor and should not be overlooked when promote mental health.

Although hobbies have been less studied [55], hobbies are described as cultivating youths’ unique areas of strength to promote positive development [56]. The present study indicated that hobby is one of the top 10 important factors in preventing depression. Hobby has the potential to enhance youth’s skills, emotion regulation and connection with peers [55].

Students may have negative emotional reactions that is understood as anxiety in face of an exam. The anxious reaction can produce physiological and behavioral changes before the exam and damage the performance during exam [57]. Some students are more likely to generate anxious reaction in exam [58]. Some students may be worried failing the exam which impacts goal achievement or let families and important others down. It is suggested to provide test preparation tips for students to reduce text anxiety. A well-prepared student is less anxious when he or she perceives that the exam is not difficult and ready for the exam [59]. Encouraging inner growth of students and making progress step by step is advised.

Adolescents is at the stage of growing quickly. Various kinds of foods not only satisfy their body needs, but may also meet their mental needs. Nutrition entered the tenth important factors. Diet rich in vegetables, fruits, berries, whole grains and fish was possibly associated with decreased risk of depression [60]. Diet with a high glycemic load increases risk of depression in participants [61]. Therefore, diet nutrition should involve various and moderate nutrient in foods. Poor nutrition may lead to low mood, and improving diet may help to protect not only the physical health but also the mental health [22].

The other study variables such as age, gender, grade, exercise, personality, self-efficacy and mindfulness did not enter the top 10 important factors. Whether their effects differ in different groups is needed to be further tested and confirmed.

The ANN also can be trained in different models that fit the characteristics of their own students. The identification risk of depression by ANN models possibly be more precise than a score model. Identifying students at high risk of depression before preventive interventions becomes very important. A good prediction or identification model for depression needs high sensitivity, specificity, and AUC. The ANN had better accuracy, sensitivity, AUC compared to other models evaluated in this study. For the study was not externally validated, whether the ANN model developed in two high schools in Eastern China performed as well in other schools was unsure. However, the ANN also can be trained in any institution for fitting the characteristics of the groups [62]. Evaluation of the use of identification models in improving the effects of preventive intervention of depression will be needed. The sensitivity is not high in the study. To improve the sensitivity, more features of variables should be included in future study. For example sleep duration, communication problems with teaching staff were recently found to be predictors of depression among health professions students [63]. In future studies, adding potential risk factors may increase sensitivity of the model. In addition, development of an instrument focused on measuring risk factors of depression may targeted depression more correctly and increase the sensitivity.

### Limitations

There were several limitations in the study. First, the study used a cross-sectional design; so, the depression had been onset in the study time not in the follow-up time. Therefore, the longitudinal association between factors and depression is needed to claim the predictive effects of ANN model in future exploration. Second, this study measured some individual factors using a single item. They may have greater survey effectiveness due to less time-consuming for participants [64]. However, single-item scales contain limited information. A standardized tool such as the dietary diversity questionnaire could have been a better option to provide more information. Third, self-report bias may exist in using the self-report measures. For personality and social psychological factors are comparatively stable variables without intervention, their valid information can be obtained from self-report measures. Finally, the study mostly focused on individual factors and social psychological factors of depression among high school students, while other important factors may exist in different groups like students with specific disease.

### Implications for practice

The findings have the following implications for further research and practice. This study employed ANN to identify risk of depression, providing the understanding of the non-linier effects of multiple factors. It implied that ANN can correctly identify students who are depressed and not depressed. Therefore, ANN is recommended in the study to identify the risk of depression of the students.

Mental health staff can target key components identified in the model in intervention program and further test and compare the effects with routine care. Improving the level of protective factors like self-compassion, optimism and resilience of students in clinical practice and school community are beneficial to decrease or prevent depression.

## Conclusions

Depression may contribute to students’ drop out, self-injury, and even suicide. To prevent this, the artificial neural network was used in the study to identify the key factors of depression and identify the students at high risk of depression. This study described the prevalence of depression among high school students and analyze the important factors contributing to depression in psychiatric nursing and offers the evaluation for the performance of the identification model. This enables a specific view on identification of risks and prevention of depression in adolescents.

## Data Availability

The datasets used and analyzed during this study are available from the corresponding author on reasonable request.

## Abbreviations

ANN: Artificial neural network
FN: False negative
FP: False positive
TN: True negative
TP: True positive
CHAID: Chi-Square Automatic Interaction Detection
C&R Tree: Classification and Regression Tree
ROC: Receiver Operating Characteristic
AUC: Area under ROC curve
